# Citronellol Potentiates Antibiotics Against *Escherichia coli* O157:H7 via Inhibition of Biofilm Formation

**DOI:** 10.3390/biom16101439

**Published:** 2026-10-02

**Authors:** Hongxing Zhang, Jing Xu, Wenkui Bao, Zhanqing Ma, Yaxin Zhou, Zhijin Zhang, Zixuan Shang, Jiyu Zhang, Yubin Bai, Bing Li

**Affiliations:** 1 Key Laboratory of New Animal Drug Project of Gansu Province, Key Laboratory of Veterinary Pharmaceutical Development of the Ministry of Agriculture and Rural Affairs, New Veterinary Drugs Engineering Research Center of Gansu Province, Key Laboratory of New Veterinary Drugs Engineering of CAAS, Laboratory Animal Science Data Center of Gansu Province, Key Laboratory of Animal Genetics and Breeding on Tibetan Plateau, Ministry of Agriculture and Rural Affairs, Lanzhou Institute of Husbandry and Pharmaceutical Sciences of CAAS, Lanzhou 730050, China; 821012420036@caas.cn (H.Z.); zhangjiyu@caas.cn (J.Z.); 2 Qinghai Provincial Agricultural Product Quality and Safety Inspection Center, Xining 810000, China; 3 College of Life Science and Food Engineering, Hebei University of Engineering, Handan 056038, China

**Keywords:** *Escherichia coli* O157:H7, biofilm, *luxS*, QS, citronellol

## Abstract

The rise in antimicrobial resistance (AMR) demands new treatments, especially for biofilm-associated infections where bacteria tolerate antibiotics. *Escherichia coli* O157:H7 (*E. coli* O157) is a virulent foodborne pathogen that causes severe haemorrhagic colitis and haemolytic uraemic syndrome. The LuxS/AI-2 quorum sensing (QS) system regulates both biofilm formation and motility in this pathogen. Here, we used a *luxS*-eGFP reporter to screen for QS modulators and identified citronellol as a potent candidate. Citronellol disrupts biofilm formation and swimming motility. These effects were accompanied by reduced *luxS* expression and modulation of *lsr* operon genes, consistent with interference with LuxS/AI-2 signalling. As a result, citronellol enhanced the antibacterial activity of conventional antibiotics at sub-MIC doses. Importantly, the citronellol–fosfomycin combination improved survival and reduced hepatic bacterial burden in a murine systemic infection model. These benefits are correlated with restored intestinal barrier integrity, including upregulated tight junction proteins (ZO-1 and Occludin). Together, these results support the continued development of citronellol-based combinations as promising anti-biofilm strategies.

## 1. Introduction

Antimicrobial resistance (AMR) represents one of the most serious global health threats. According to the World Health Organization, bacterial AMR was associated with more than 4.7 million deaths worldwide in 2021, including approximately 1.14 million deaths directly caused by resistant infections [1]. In 2023, approximately one in six lab-confirmed bacterial infections were resistant, and resistance increased in more than 40% of pathogen and antibiotic combinations monitored from 2018 to 2023 [2]. The therapeutic efficacy of conventional antibiotics is increasingly compromised not only by the emergence of resistant planktonic bacteria but also by the ability of pathogens to form structured communities known as biofilms [2]. Biofilm-associated infections represent a major contributor to chronic clinical conditions, such as cystic fibrosis pneumonia, chronic wounds, and implant-related infections [3]. These infections are notoriously difficult to eradicate because biofilm-embedded bacteria exhibit extreme tolerance to antibiotics and avoid host defenses [4]. Within biofilms, bacteria are encased in a self-produced extracellular polymeric substance matrix that restricts antibiotic penetration, fosters a slow-growth phenotype, and promotes horizontal gene transfer, which collectively contributes to tolerance levels 10–1000 times higher than those of planktonic bacteria [5]. Crucially, this coordinated behavior is orchestrated by quorum sensing (QS), a chemical signaling mechanism that enables bacteria to sense population density and collectively regulate biofilm maturation and host colonization [6]. In multiple pathogens, QS controls extracellular matrix production, microcolony development, and dispersal, whereas the LuxS/AI-2 signaling plays a key role in early biofilm assembly and mucosal colonization [7,8]. Proteomic and docking evidence from related species further suggest the LuxS/AI-2 axis as a potential target [9,10]. Accordingly, we employed a *luxS*-eGFP reporter system to screen our natural compound library and identified citronellol as a candidate that reduced luxS-driven fluorescence, leading to its selection for further investigation. Citronellol is an acyclic monoterpene alcohol (C_10_H_20_O, MW 156.27 g/mol) predominantly found in Cymbopogon spp. (lemongrass), rose oils, and geranium. With a calculated logP of ~3.1, it is highly lipophilic, enabling passive diffusion across bacterial membranes and interaction with hydrophobic signaling molecules [11]. Beyond its established QS inhibition and biofilm-disrupting activities, citronellol exhibits broad biological effects, including antimicrobial, anti-inflammatory, and antioxidant properties [12,13,14]. These properties make citronellol a promising candidate against serious foodborne pathogens such as *Escherichia coli* O157:H7 (*E. coli* O157).

*E. coli* O157 is a common worldwide pathogen causing millions of cases of severe diarrheal disease annually [15]. Its LuxS/AI-2–dependent biofilm phenotype underpins environmental persistence and contamination across the food chain [16], shielding the pathogen from host immune responses and acting as a reservoir for antimicrobial-resistant strains [17,18]. Moreover, recurrent contamination along the food production chain significantly increases the risk of f severe infection [19,20]. Consequently, disrupting biofilm formation is a key strategy to reduce both the environmental reservoir and clinical burden of *E. coli* O157. Within this QS network, the LuxS/AI-2 pathway orchestrates *E. coli* O157 biofilm development [21,22]. Thus, intercepting the LuxS/AI-2 axis disrupts biofilm initiation, preventing the assembly of a physical barrier and rendering bacteria more susceptible to antimicrobial attack while reducing resistance selection [23,24]. We hypothesized that citronellol impairs *E. coli* O157 biofilm formation and enhances antibiotic susceptibility through modulation of the LuxS/AI-2 signaling axis. To test this, we combined a *luxS*-eGFP reporter assay with phenotypic evaluations of QS inhibition, biofilm architecture, and metabolic activity, followed by assessment of citronellol–antibiotic combinations in a murine *E. coli* O157 infection model (fosfomycin sodium + citronellol). Collectively, these results show citronellol as an effective biofilm-disrupting adjuvant that potentiates conventional antibiotics against refractory biofilm-associated infections while reducing the risk of resistance emergence.

## 2. Materials and Methods

### 2.1. Materials and Bacteria

The citronellol (≥98% purity, CAS Number: 106-22-9) used in this study was purchased from MedChemExpress Co., Ltd. (Shanghai, China; Catalogue No. HY-W010201). The chemical structure of citronellol is shown in Supplementary Figure S1. Citronellol stock solutions were prepared in dimethyl sulfoxide (DMSO; Sigma-Aldrich, Burlington, MA, USA; Catalogue No. D2650) and stored at −20 °C until use. The final concentration of DMSO in all assays did not exceed 1% (*v*/*v*). For control experiments, an equal volume of DMSO without the compound was added to account for the solvent effect. Polymyxin B (≥98%), tetracycline hydrochloride (≥98%), fosfomycin sodium (≥98%), gentamicin sulfate (≥98%), azithromycin (≥98%), cefotaxime acid (≥98%), chloramphenicol (≥98%), and streptomycin (≥98%) were purchased from Shanghai Yuanye Biotechnology Co., Ltd. (Shanghai, China). All antibiotics were of analytical grade or higher and used without further purification.

*E. coli* O157 (ATCC 43895) was obtained from the American Type Culture Collection (ATCC, Manassas, VA, USA). *Vibrio harveyi* BB170 and BB152, used for bioluminescence assays, were kind gifts from Prof. Xiang’an Han (Shanghai Veterinary Research Institute, Chinese Academy of Agricultural Sciences, Shanghai, China). Luria–Bertani (LB) broth and agar (Huankai Microbial Sci. & Tech. Co., Ltd., Guangzhou, China) were used for routine cultivation. For *V. harveyi* assays, bacteria were cultured in AB medium (0.05 M MgSO_4_, 0.3 M NaCl, and 0.2% vitamin-free Casamino Acids) supplemented with 1 mM L-arginine, 1% phosphate buffer (pH 7.2), and 1% glycerol.

### 2.2. LuxS-eGFP Reporter Strain Construction

Genomic DNA was extracted from *E. coli* O157 using the TIANamp Bacteria DNA Kit (DP302; Tiangen Biotech, Beijing, China) according to the manufacturer’s protocol. The *luxS* gene was amplified from the genomic DNA using primers *luxS*-F and *luxS*-R (Supplementary Table S1), which were designed to incorporate EcoRI and BamHI restriction sites at the 5′ and 3′ ends. PCR was performed with PrimeSTAR Max DNA Polymerase (Takara Bio Inc., Otsu, Shiga, Japan; Cat. No. R045A) in 2× Premix buffer following the manufacturer’s protocol, with initial denaturation at 98 °C for 3 min, 30 cycles of 98 °C for 10 s, 55 °C for 15 s, and 72 °C for 30 s. The eGFP fragment was amplified from a plasmid containing the eGFP gene using primers eGFP-F and eGFP-R (Supplementary Table S1). The purified *luxS* amplicon and *eGFP* fragment were fused via overlap extension PCR using primers luxS-F and eGFP-R. Equimolar amounts of the two fragments were mixed and subjected to an initial extension step without primers to allow annealing of the overlapping regions, followed by amplification with the outer primers to generate the *luxS*-eGFP fusion product. The purified *luxS*-eGFP fusion product was double-digested with EcoRI and BamHI (Takara Bio Inc.) at 37 °C for 3 h, and the pUC-19 vector was similarly digested at the corresponding sites. The digested fragments were ligated into pUC-19 using T4 DNA ligase (Takara Bio Inc., Cat. No. 2011B) following the manufacturer’s protocol. The schematic map of the plasmid construct is provided in Supplementary Figure S3. The ligation product was transformed into *E. coli* DH5α competent cells by heat shock, and transformants were selected on LB agar containing 100 μg/mL ampicillin. Positive clones were identified by colony PCR and confirmed by Sanger sequencing. The validated construct was then electroporated into *E. coli* O157 competent cells to generate the *luxS*-eGFP reporter strain.

### 2.3. LuxS/AI-2 Pathway Screening

The *luxS-eGFP* reporter strain was cultured at 37 °C with shaking (200 rpm) until the optical density at OD_600_ reached 0.6. Protein expression was induced by the addition of isopropyl-*β*-D-thiogalactopyranoside (IPTG) to a final concentration of 1 mM, followed by a 4-h incubation. Subsequently, the cultures were treated with the quorum-sensing inhibitor furanone C-30 (Sigma-Aldrich, Burlington, MA, USA) [25,26] at a final concentration of 60 μM (diluted into the culture to a final DMSO concentration of 0.1% *v*/*v*). After a 4-h incubation at 37 °C with shaking, fluorescence was measured. The untreated *luxS*-eGFP-expressing culture served as the positive control (maximum fluorescence signal), and the wild-type *E. coli* O157 strain without the eGFP construct served as the negative control (background autofluorescence) for Z’ factor calculation. From three independent experiments, the positive control gave a mean fluorescence of 19,947 ± 198, and the negative control gave 479 ± 94. The Z’ factor was calculated according to the standard formula of Zhang, et al. [27]. These values yielded a value of 0.955, confirming the robustness of the screening platform. For small-molecule screening, test compounds were added simultaneously with IPTG. After a 4-h incubation, 200 μL of each culture was transferred to a black opaque 96-well plate (in triplicate). Fluorescence intensity was quantified using a multimode microplate reader (excitation: 488 nm; emission: 509 nm). The assay was performed in three independent biological replicates. Compounds exhibiting significant inhibition of *luxS-eGFP* fluorescence were selected for subsequent experiments.

Single colonies of *E. coli* were inoculated into LB broth and subcultured twice. The cultures were adjusted to OD_600_ = 1.0 and diluted 1:100 into fresh LB broth supplemented with the *luxS* inhibitor or test compounds, followed by incubation for 16 h. After centrifugation at 12,000× *g* for 5 min, the supernatant was sterile-filtered through a 0.22-µm membrane to obtain the cell-free supernatant (CFS) [28].

*V. harveyi* BB170 was cultured in AB medium and subcultured twice. The culture was then diluted 1:100 into fresh AB medium and incubated at 30 °C with shaking (160 rpm) until the OD_600_ reached approximately 1.0 [23]. Subsequently, the cells were diluted 1:2500 in AB medium. For the AI-2 bioassay, 20 μL of CFS from *E. coli* cultures was mixed with 180 μL of the diluted *V. harveyi* BB170 suspension in a white opaque 96-well plate. After incubation in the dark at 30 °C for 4 h, bioluminescence intensity was measured using a multimode microplate reader. CFS from an overnight culture of *V. harvey Yi BB152* served as the positive control, and all assays were performed in triplicate.

### 2.4. qRT-PCR

The effect of citronellol on the transcriptional profiles of quorum sensing and biofilm-related genes in *E. coli* O157 was evaluated via quantitative real-time PCR (qRT-PCR). Total RNA was isolated from *E. coli* O157 cultures exposed to 200 µg/mL citronellol for 24 h at 37 °C using the Bacterial RNA Kit (Omega, Norcross, GA, USA). RNA integrity and concentration were assessed using a NanoDrop OneC spectrophotometer (Thermo Scientific, Waltham, MA, USA). Subsequently, residual genomic DNA was eliminated, and reverse transcription was performed using the PrimeScript™ RT Reagent Kit with gDNA Eraser (Takara, Kusatsu, Japan). Gene expression levels were quantified using TB Green^®^ Premix Ex Taq™ II (TAKARA, Kusatsu, Japan), using the 16S rRNA gene as an internal reference due to its stable transcription. Relative transcriptional changes were calculated using the 2^−∆∆Ct^ method. All qPCR reactions were performed in triplicate using the primers listed in Supplementary Table S2.

### 2.5. Minimum Inhibitory Concentration (MIC)

The two-fold serial broth microdilution method was used to determine the minimum inhibitory concentration (MIC) of citronellol against *E. coli* O157 in a 96-well plate. Briefly, Mueller–Hinton broth containing 0.15% (*w*/*v*) agar (MHBA) was prepared, and citronellol concentrations ranged from 400 to 6.25 μg/mL. Overnight *E. coli* O157 cultures were diluted to approximately 1 × 10^6^ CFU/mL in MHBA. Each well of the 96-well plate contained 100 μL of MHBA and 100 μL of the bacterial suspension (1 × 10^6^ CFU/mL), resulting in a final inoculum of approximately 5 × 10^5^ CFU/mL per well. Positive control wells contained *E. coli* O157 (5 × 10^5^ CFU/mL) in MHBA without citronellol. Wells containing 200 μL of uninoculated MHBA served as blanks. The plates were incubated statically at 37 °C for 24 h. The MIC was defined as the lowest concentration of citronellol at which no visible bacterial growth (i.e., no change in turbidity) was observed. All experiments were performed in triplicate.

### 2.6. Bacterial Growth and Viability Assays

The effect of citronellol on the growth and viability of *E. coli* O157 was monitored using CFU (colony-forming unit) assays. Briefly, overnight cultures were diluted 1:100 into fresh LB medium containing citronellol at final concentrations ranging from 50 to 200 µg/mL. The cultures were incubated at 37 °C with continuous shaking. To evaluate growth kinetics, aliquots were collected from each treatment group at designated time points (0, 2, 4, 8, and 12 h), serially diluted in sterile PBS, and plated onto LB agar. Colonies were counted after incubation at 37 °C for 24 h. Bacterial cultures without citronellol served as negative controls. All experiments were performed in triplicate.

### 2.7. Biofilm Inhibition

Biofilm inhibition assays were performed using the crystal violet (CV) staining assay [29]. *E. coli* O157 was initially cultured at 37 °C for 24 h. Following this, the bacterial suspension was standardised to an OD_600_ of 0.01 and dispensed into white 96-well plates (Corning Costar^®^ 3599, Corning, Kennebunk, ME, USA) containing citronellol at concentrations ranging from 6.25 to 400 µg/mL. Following a 24-h incubation at 37 °C, non-adherent cells were removed by three washes with PBS (pH 7.2). The remaining biofilms were fixed with 95% ethanol for 15 min at room temperature, then stained with 0.1% (*w*/*v*) crystal violet for 30 min at room temperature. Excess dye was removed by rinsing three times with PBS. The bound dye was solubilised with 33% (*v*/*v*) glacial acetic acid (150 μL per well) for 15 min at room temperature, and the absorbance was measured at 595 nm using a multimode microplate reader. The amount of CV retained by the biofilms was calculated as the mean OD595 value of triplicate wells, with background subtraction from blank wells containing medium only (no bacteria). Biofilm inhibition was expressed as the percentage reduction in OD595 relative to the untreated control.

### 2.8. Metabolic Activity

The metabolic activity of *E. coli* O157 was assessed using the Alamar Blue assay [24] following exposure to citronellol concentrations ranging from 12.5 to 400 μg/mL. Briefly, cells were harvested from 48-well plates into fresh microcentrifuge tubes, centrifuged, washed with PBS, and resuspended in 1 mL of PBS. Aliquots of the cell suspension were then transferred to a 96-well plate, followed by the addition of 10 μL of Alamar Blue dye. After incubation at 37 °C in the dark for 1 h, absorbance was measured at 570 nm and 600 nm using a microplate reader. A blank control consisting of PBS with dye only was included to calculate metabolic activity based on the following formula:

$$\mathrm{Metabolic}\;\mathrm{activity}\;(\%)=\frac{{((E}_{\mathrm{oxi}(\mathrm{OD}600)}\times T_{\mathrm{OD}570})-{(E}_{\mathrm{oxi}\left(\mathrm{OD}570\right)}\times T_{\mathrm{OD}600}))}{{((E}_{\mathrm{red}(\mathrm{OD}570)}\times B_{\mathrm{OD}600})-{(E}_{\mathrm{red}(0D600)}\times B_{\mathrm{OD}570}))}\times 100\%$$

Eoxi_(OD570)_—extinction coefficient of AB in its oxidized form at 570 nm = 80,586;

Ered_(OD570)_—extinction coefficient of AB in its reduced form at 570 nm = 155,677;

Eoxi_(OD600)_—extinction coefficient of AB in its oxidized form at 600 nm = 117,216;

Ered_(OD600)_—extinction coefficient of AB in its reduced form at 570 nm = 14,652;

B—blank; T—samples.

### 2.9. Scanning Electron Microscopy (SEM) Analysis

Biofilm morphology was examined via scanning electron microscopy (SEM) with established protocols [30]. Briefly, bacterial suspensions were inoculated into 96-well plates containing sterile coverslips and exposed to 50–200 µg/mL citronellol, with an untreated bacterial suspension serving as the control. Following a 24-h incubation at 37 °C to facilitate biofilm formation, the supernatant was removed and the coverslips were gently washed three times with sterile PBS (pH 7.4) to dislodge planktonic cells and loosely attached debris. The coverslips were then carefully removed and fixed by full immersion in 2.5% (*v*/*v*) glutaraldehyde solution at 4 °C for 12 h. After fixation, samples were washed with PBS and dehydrated through a graded ethanol series (30%, 50%, 80%, 90%, and 100% ethanol, 15 min per step), followed by critical point drying. The dried samples were mounted on aluminum stubs and sputter-coated with gold. Finally, samples were imaged using an FEI Versa 3D SEM (Thermo Fisher Scientific, Waltham, MA, USA) at an accelerating voltage of 5 kV and a magnification of ×5000.

### 2.10. Bacterial Motility

The impact of citronellol on *E. coli* O157 motility was assessed using a semi-solid agar assay [31]. Briefly, an overnight culture was standardised to an OD_600_ of 1.0. Subsequently, 1 μL of the bacterial suspension was spotted onto 0.3% semi-solid LB agar plates supplemented with varying concentrations of citronellol (50–200 µg/mL). Following incubation at 37 °C for 24 h, motility was quantified by measuring the migration halo diameter relative to the control. Each experiment was performed in triplicate.

### 2.11. Combination Antibacterial Activity

The minimum inhibitory concentrations (MICs) of various antibiotics against *E. coli* O157 were determined in accordance with the Clinical and Laboratory Standards Institute (CLSI) document M07, “Methods for Dilution Antimicrobial Susceptibility Tests for Bacteria That Grow Aerobically”. The MIC values were as follows: Azithromycin (0.25 µg/mL), Polymyxin B (1 µg/mL), Cefotaxime acid (0.0625 µg/mL), Streptomycin (2 µg/mL), Chloramphenicol (4 µg/mL), Gentamicin (8 µg/mL), and Tetracycline (0.5 µg/mL). The MIC of fosfomycin Sodium was found to be 8 µg/mL. The combined activity of citronellol with conventional antibiotics was evaluated following Bai, et al. [32]. Briefly, an overnight culture of *E. coli* O157 was diluted to an OD_600_ of 0.01 and exposed to sub-inhibitory concentrations of antibiotics (1/2, 1/4, and 1/8 MIC) with or without citronellol (200 µg/mL). Following a 16- to 18-h incubation at 37 °C, metabolic activity was quantified using the Alamar Blue assay. The combined effect was classified based on statistical comparison of viability between the combination group and the antibiotic-only group at the same concentration. The tests were performed in triplicate (*n* = 3).

### 2.12. In Vivo Efficacy of Antibiotic–Citronellol Combinations

The Female 6-week-old C57BL/6J mice (18–22 g) were procured from the Lanzhou Veterinary Research Institute, Chinese Academy of Agricultural Sciences. All animal procedures were conducted in strict accordance with the guidelines for the care and use of laboratory animals and were approved by the Institutional Animal Care and Use Committee of the Lanzhou Institute of Husbandry and Pharmaceutical Sciences of CAAS (Approval No. 2025-16). Following a 3-day acclimatisation period, mice were randomised into four groups (*n* = 12 per group): (1) untreated control, (2) fosfomycin sodium alone, (3) citronellol alone, and (4) combination therapy (citronellol + fosfomycin sodium). *E. coli* O157 was cultured overnight, subcultured once, and adjusted to a 0.5 McFarland standard. The suspension was then centrifugally concentrated to a final density of 1.5 × 10^9^ CFU/mL. On day 1, mice were intraperitoneally challenged with 0.2 mL/10 g of the bacterial suspension. One hour post-infection, treatments were administered orally. Mice in the fosfomycin and combination groups received fosfomycin sodium (2.5 mg/mL) with or without citronellol (100 mg/kg), based on prior murine studies reporting efficacy without acute toxicity [33,34,35]. Survival was recorded daily over a 7-day period, and Kaplan–Meier survival curves were generated. On day 7, surviving mice were euthanized by cervical dislocation following induction with isoflurane anaesthesia (RWD Life Science Co., Ltd., Shenzhen, China). Spleen, liver, and ileum tissues were harvested, fixed in 4% paraformaldehyde (Wuhan Servicebio Technology Co., Ltd., Wuhan, China), dehydrated through a graded ethanol series, cleared in xylene, embedded in paraffin wax, and sectioned at 5–8 µm using a rotary microtome. Sections were stained with haematoxylin and eosin (H&E) for histopathological evaluation [36]. Bacterial loads in target tissues were quantified using standard plate counting methods.

### 2.13. Immunofluorescence, Hematoxylin, and Eosin

Liver, spleen, and ileum tissues were collected and fixed in 4% paraformaldehyde (Wuhan Servicebio Technology Co., Ltd., Wuhan, China) overnight. The tissues were then dehydrated through graded ethanol, cleared in xylene, and embedded in molten paraffin. Serial sections (5–8 μm thick) were cut using a microtome. For histopathological analysis, the sections were deparaffinized, rehydrated, and stained with hematoxylin and eosin (H&E) for microscopic examination. For immunofluorescence staining of ileal tight junction proteins, the sections were deparaffinized, rehydrated, and subjected to antigen retrieval if necessary. After washing with PBS, the sections were blocked with bovine serum for 30 min to reduce background staining. The sections were then incubated overnight at 4 °C with primary antibodies against ZO-1 and occludin. Following three washes, the sections were incubated with the corresponding fluorescently labeled secondary antibody in the dark at room temperature for 1 h. Finally, the nuclei were counterstained with DAPI, and the sections were mounted with antifade mounting medium for fluorescence imaging.

### 2.14. Statistical Analysis

All statistical evaluations and graphical representations were generated using GraphPad Prism (version 10.0, GraphPad Software, San Diego, CA, USA). Data normality and homogeneity of variances were assessed prior to analysis. Data are presented as mean ± standard deviation (SD) of *n* independent biological replicates. For experiments involving multiple groups, statistical significance was assessed using a Kruskal–Wallis test followed by Dunn’s post hoc test for multiple comparisons. For comparisons between two groups, an unpaired *t*-test was used. For survival analysis, Kaplan–Meier curves were constructed and compared using the log-rank (Mantel–Cox) test. Exact *p* values are provided in the figures, tables, and text. Statistical significance was defined as *p* < 0.05.

## 3. Results

### 3.1. Modulation of LuxS-Mediated Signaling by Citronellol

**Figure 1:**
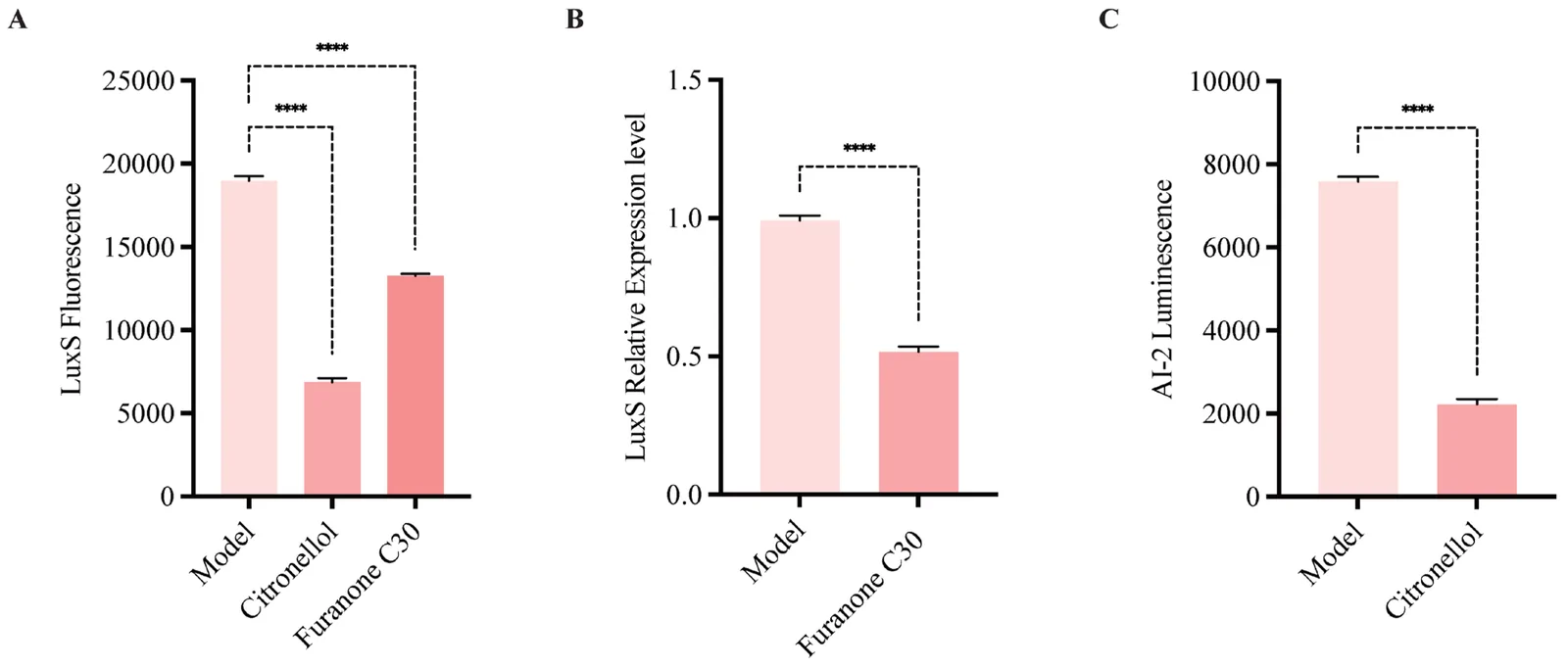
Inhibitory effects of citronellol and furanone C30 on the *luxS*-eGFP reporter system. (**A**) Fluorescence of the *luxS*-eGFP reporter strain following treatment with citronellol or furanone C30. (**B**) Relative expression level of the *luxS* gene in the *luxS-eGFP* reporter strain treated with Furanone C30. (**C**) The AI-2 activity assessed via bioluminescence of the *V. harveyi* BB170 reporter strain following citronellol treatment. (*n* = 3 per group; data represent means ± SD; **** *p* < 0.0001.)

To validate the functionality of the constructed *luxS-eGFP* reporter system, the known QS inhibitor furanone C30 was used as a positive control. As shown in Figure 1A, treatment with furanone C30 significantly suppressed *luxS*-eGFP strain fluorescence intensity. Notably, citronellol treatment also reduced fluorescence output in this assay (Figure 1A, *p* < 0.0001). Furthermore, quantitative analysis showed lower *luxS* transcription, as furanone C30 markedly downregulated the relative expression level of the *luxS* gene in the *luxS*-eGFP strain (Figure 1B, *p* < 0.0001). To further elucidate citronellol effects on QS signalling, AI-2 activity was assessed using the biosensor strain *V. harveyi* BB170. Citronellol treatment decreased AI-2-mediated bioluminescence in the *V. harveyi* BB170 reporter assay compared to the untreated control (Figure 1C, *p* < 0.0001). Collectively, these results demonstrate that citronellol reduces fluorescence output in the *luxS*-eGFP reporter assay. It also decreases AI-2-mediated bioluminescence in the *V. harveyi* BB170 reporter system.

### 3.2. Citronellol Disrupts Biofilm Formation and Motility in E. coli O157

**Figure 2:**
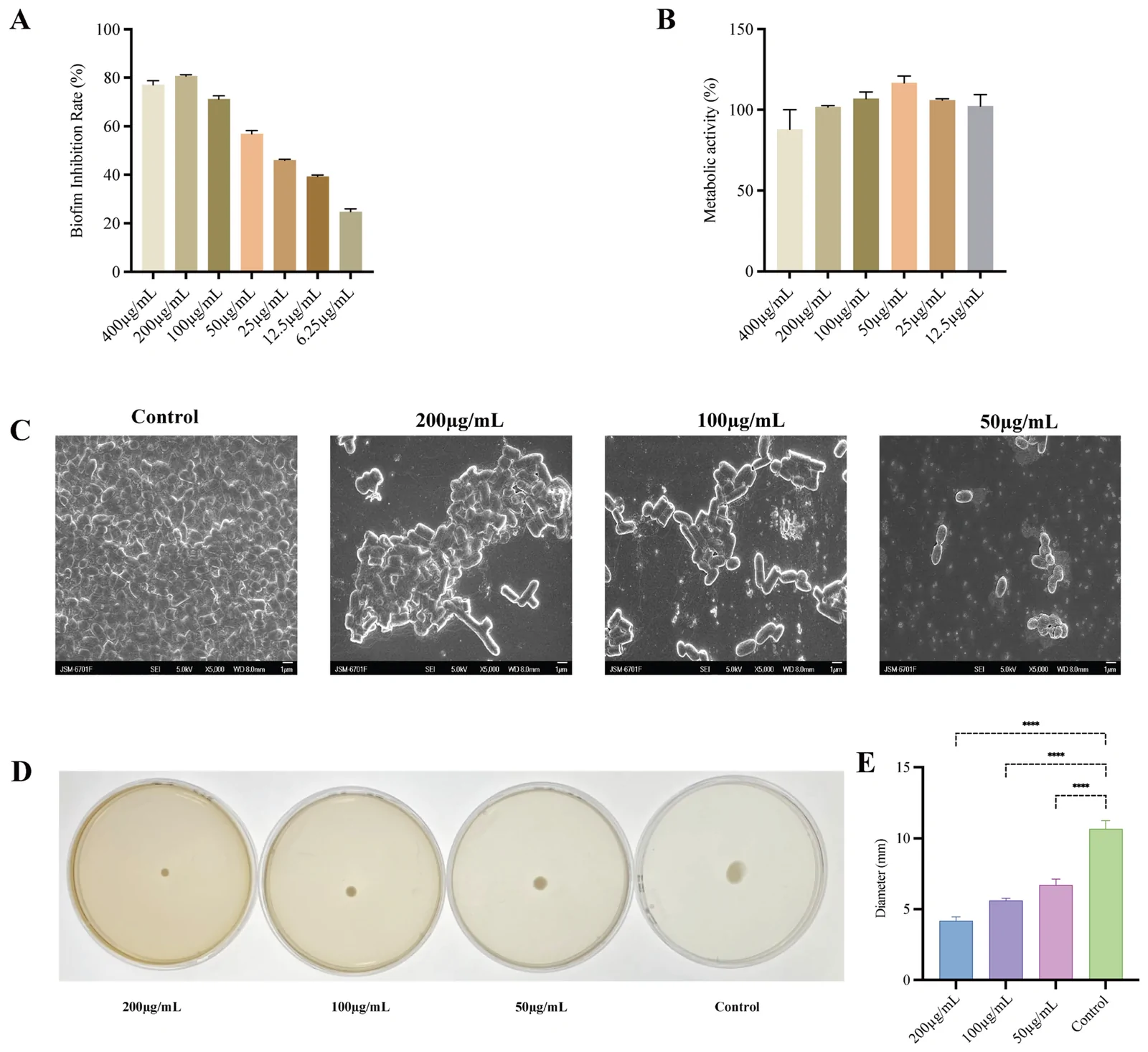
Citronellol inhibits biofilm formation and motility in *E. coli O157*. (**A**) Biofilm inhibition rate of *E. coli* O157 after treatment with increasing concentrations of citronellol. (**B**) Metabolic activity of *E. coli* O157 after treatment with citronellol. (**C**) Scanning electron micrographs of *E. coli* O157 treated with different concentrations of citronellol (scale bar = 1 μm). (**D**) Representative images of swimming motility assays. (**E**) Quantification of swimming diameters, indicating a dose-dependent inhibition of *E. coli* O157 swimming motility by citronellol. (*n* = 3 per group; data represent means ± SD; **** *p* < 0.0001.)

Citronellol showed no direct antibacterial activity against *E. coli* O157:H7 (MIC > 400 μg/mL), confirming that all tested concentrations (6.25–400 μg/mL) were sub-inhibitory. Nevertheless, quantitative analysis of biofilm inhibition revealed a robust dose-dependent response to citronellol treatment (Figure 2A). Total biofilm biomass decreased in a dose-dependent manner up to 200 µg/mL, peaking at approximately 80%. Beyond this concentration, the inhibitory effect plateaued, remaining stable at 400 µg/mL, indicating that 200 µg/mL was the best suppressive dose. Notably, even at the lowest tested concentration of 6.25 µg/mL, citronellol retained substantial biofilm inhibitory activity, achieving a suppression rate over 20%. Importantly, this reduction in biofilm biomass was not associated with impaired bacterial growth or metabolic activity. Metabolic activity assays (Figure 2B) confirmed that citronellol did not substantially alter metabolic activity across the tested concentrations (*p* > 0.05 vs. untreated control). Consistently, CFU-based growth kinetics over 12 h (Supplementary Figure S2) revealed no significant difference between the citronellol-treated and control groups, indicating that the anti-biofilm effect is not a consequence of growth inhibition. Scanning electron microscopy provided qualitative ultrastructural visualization of these phenotypic changes (Figure 2C). While the model group exhibited dense, compact multilayer biofilms, citronellol treatment induced significant architectural disruption. At 50 and 100 µg/mL, a noticeable reduction in cell density and increased intercellular spacing were observed. At 200 µg/mL, the biofilm matrix was markedly broken down, showing mostly isolated cells and small clusters attached to the substrate. In addition to suppressing biofilm formation, citronellol also significantly com impaired planktonic motility. Swimming motility assays (Figure 2D) revealed a dose-dependent restriction of bacterial migration. While the control group showed extensive spreading across the agar surface, citronellol treatment gradually reduced the swimming zone, leading to a much smaller area of spread. Quantification of swimming diameters (Figure 2E) substantiated this visual observation, revealing a significant, dose-dependent reduction in swimming motility (*p* < 0.0001). Collectively, these results demonstrate that citronellol effectively suppresses key virulence-related phenotypes in *E. coli* O157. It disrupts both biofilm formation and motility.

### 3.3. Citronellol Modulates AI-2 QS and Motility Genes in E. coli O157

**Figure 3:**
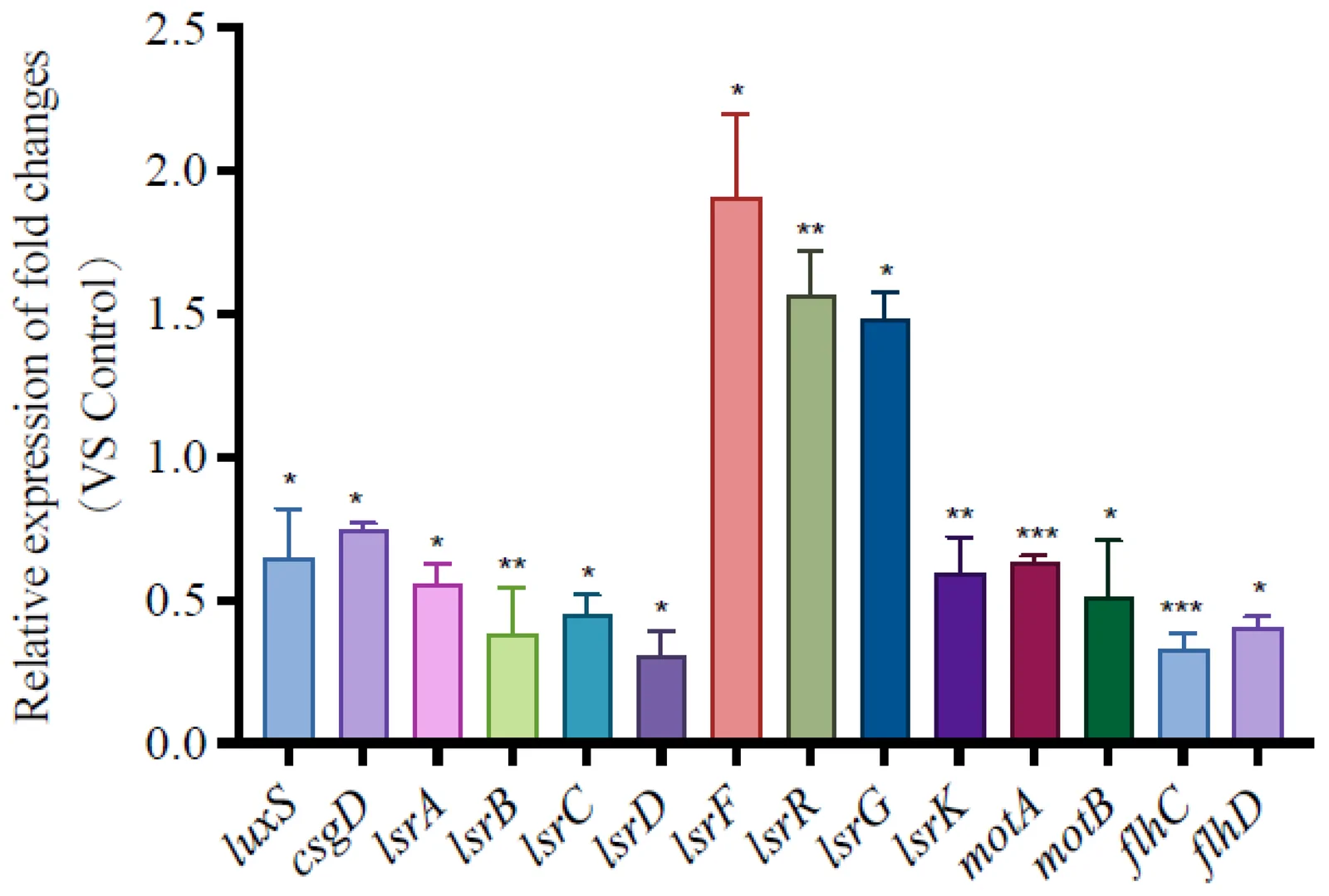
Relative mRNA expression levels of the core AI-2 system genes, including the synthase *luxS* and the *lsr* operon components (*lsrA*, *lsrB*, *lsrC*, *lsrD*, *lsrF*, *lsrG*, *lsrK*, *lsrR*), as well as the biofilm-associated regulator *csgD* and flagellar motility genes (*flhC*, *flhD*, *motA*, *motB*), after treatment with citronellol. (*n* = 3 per group; data represent means ± SD; * *p* < 0.05, ** *p* < 0.01, *** *p* < 0.001.)

After observing the strong inhibitory effects of citronellol on biofilm formation and motility, we subsequently investigated the transcriptional alterations associated with these phenotypic changes. As shown in Figure 3, citronellol changed the expression of luxS/AI-2 genes and virulence regulators (*lsr* operon, *csgD*, *flhC*, *flhD*, *motA*, and *motB*). Treatment with citronellol resulted in a significant, coordinated upregulation of the *lsr* operon components. Specifically, *lsrF*, *lsrR*, and *lsrG* expression levels were markedly elevated, showing approximately 1.8-, 1.5-, and 1.4-fold increases (all *p* < 0.05). Conversely, the AI-2 importer genes *lsrA*, *lsrB*, *lsrC*, *lsrD*, and *lsrK* were significantly downregulated (ranging from 0.3- to 0.6-fold, all *p* < 0.05). Consistently, the AI-2 synthase gene *luxS* was significantly downregulated after citronellol treatment (approximately 0.65-fold, *p* < 0.05). In addition, citronellol affected many genes involved in bacterial motility and biofilm architecture. The expression of *csgD*, which serves as the master regulator of biofilm matrix production, was significantly repressed (approximately 0.45-fold, *p* < 0.05). Citronellol also suppressed flagellar assembly genes, including the transcription factors *flhC* and *flhD* (both <0.4-fold, *p* < 0.05) and the motor proteins *motA* and *motB* (approximately 0.5-fold, *p* < 0.001 and *p* < 0.05, respectively).

### 3.4. Combination Effects of Citronellol with Antibiotics

**Figure 4:**
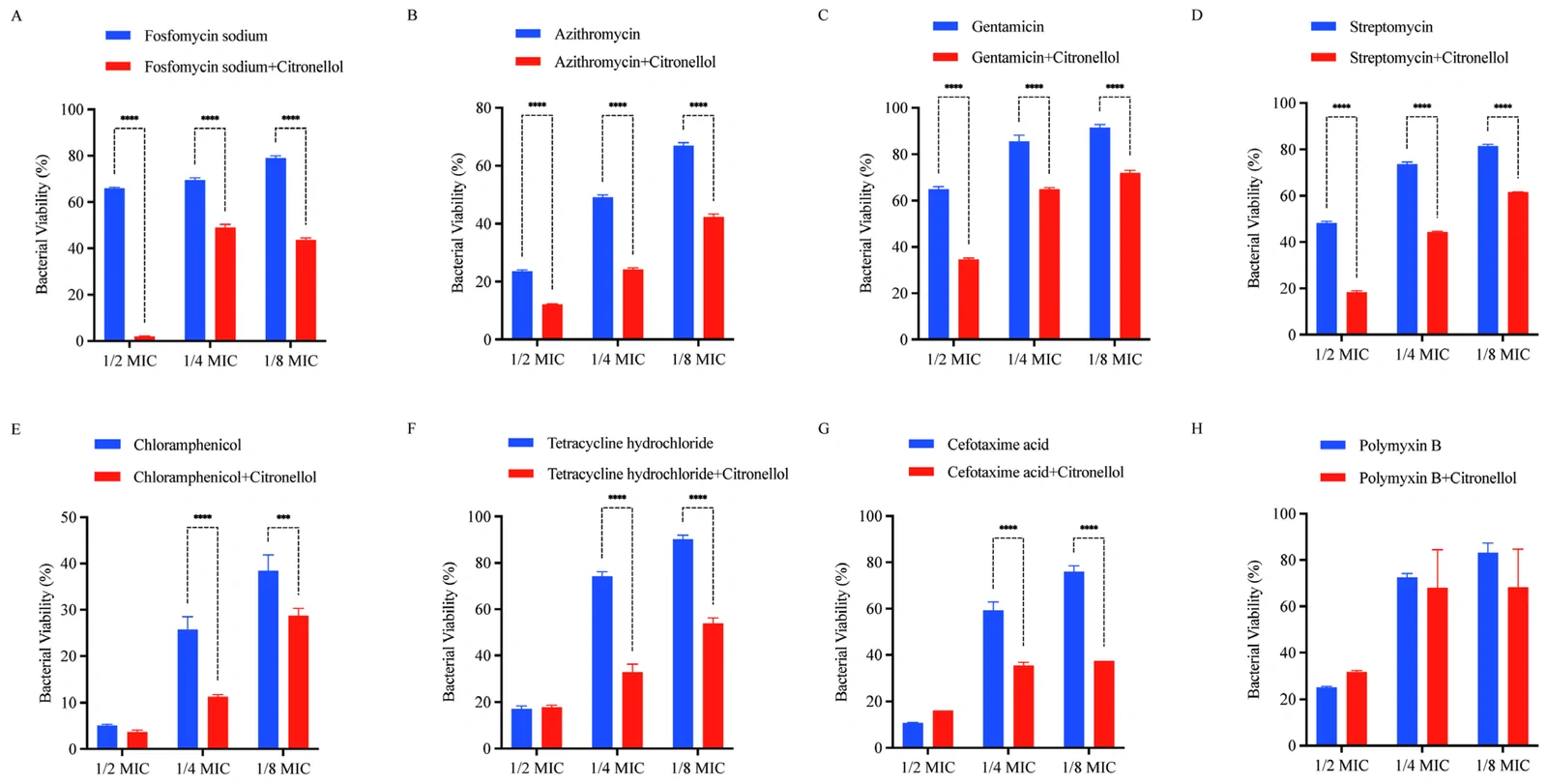
Effects of antibiotic–citronellol combination on bacterial viability. Bar graphs (**A**–**H**) show the viability of *E. coli* O157 treated with eight common antibiotics (fosfomycin sodium, azithromycin, gentamicin, streptomycin, chloramphenicol, tetracycline hydrochloride, cefotaxime acid, and polymyxin B) at sub-inhibitory concentrations (1/2, 1/4, and 1/8 MIC), either alone or in combination with a fixed concentration of citronellol. (*n* = 3 per group; data represent means ± SD; *** *p* < 0.001, **** *p* < 0.0001.)

As shown in Figure 4, we evaluated the combination effects of citronellol with eight conventional antibiotics against *E. coli* O157 at sub-inhibitory concentrations (1/2, 1/4, and 1/8 MIC). As individual antibiotics, these drugs exhibited variable efficacy, with viability rates ranging from 10% to 90%. However, combining citronellol with these antibiotics significantly augmented the antibacterial activity against *E. coli* O157. Marked potentiation was observed with fosfomycin sodium (Figure 4A), azithromycin (Figure 4B), and streptomycin (Figure 4D). For instance, citronellol reduced bacterial viability to below 30% at 1/2 MIC (from ~65%, 20%, and 50% with antibiotics alone, respectively). This effect continued at lower doses, but the benefit decreased as antibiotic doses were reduced. Similarly, gentamicin (Figure 4C) combined with citronellol led to bacterial viability below 40% at 1/2 MIC and remained significantly lower than monotherapy at 1/4 and 1/8 MIC. In contrast, chloramphenicol (Figure 4E), tetracycline hydrochloride (Figure 4F), and cefotaxime acid (Figure 4G), showed no significant effect at 1/2 MIC. However, the combination of citronellol and antibiotics increased antibacterial activity at 1/4 and 1/8 MIC. No potentiation was observed with polymyxin B (Figure 4H) at any sub-inhibitory concentration. Together, these data demonstrate that citronellol acts as an effective antimicrobial adjuvant, significantly augmenting the inhibitory effects of diverse antibiotic classes at sub-MIC doses.

### 3.5. Protective Efficacy of the Combination of Citronellol and Fosfomycin Sodium

**Figure 5:**
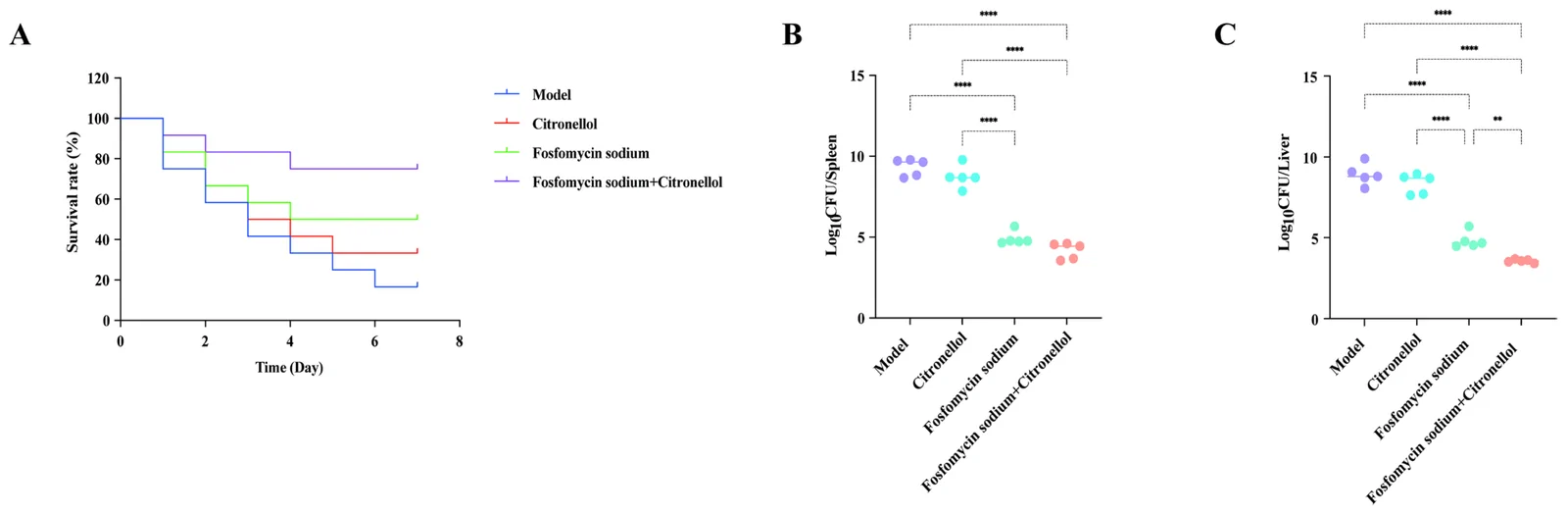
The efficacy of citronellol in combination with fosfomycin sodium against *E. coli* O157 infection in a murine model. (**A**) Survival rate of mice following *E. coli* O157 infection under different treatments. Mice (*n* = 12 per group) were treated with vehicle (Model), citronellol alone, fosfomycin sodium alone, or their combination. (**B**) Bacterial loads in the spleen of *E. coli* O157-infected mice after different treatments. Mice were treated with vehicle (Model), citronellol alone, fosfomycin sodium alone, or their combination. (**C**) Bacterial loads in the liver of E. coli O157-infected mice after different treatments. (*n* = 3 per group; data represent means ± SD; ** *p* < 0.01, **** *p* < 0.0001.)

**Figure 6:**
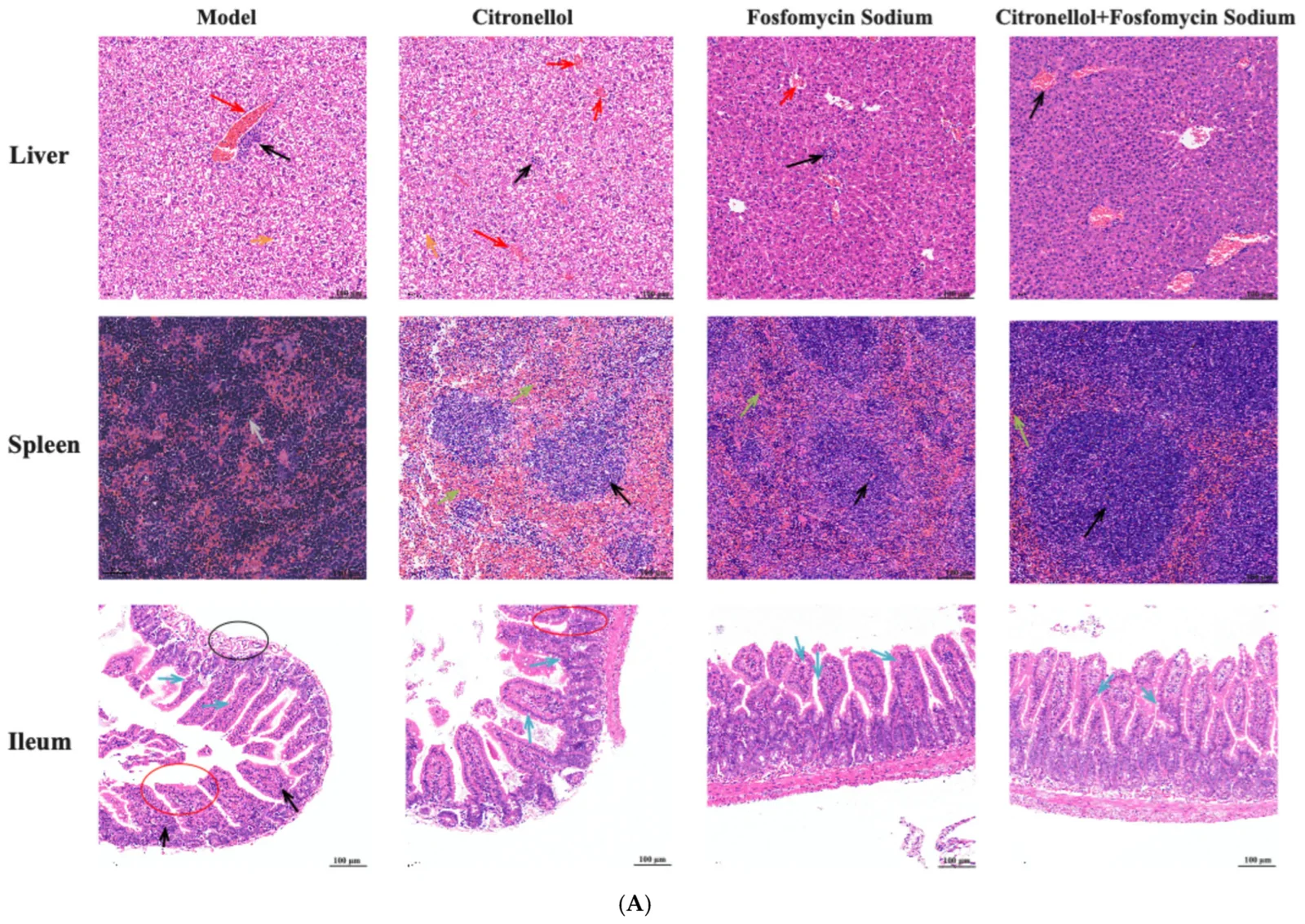

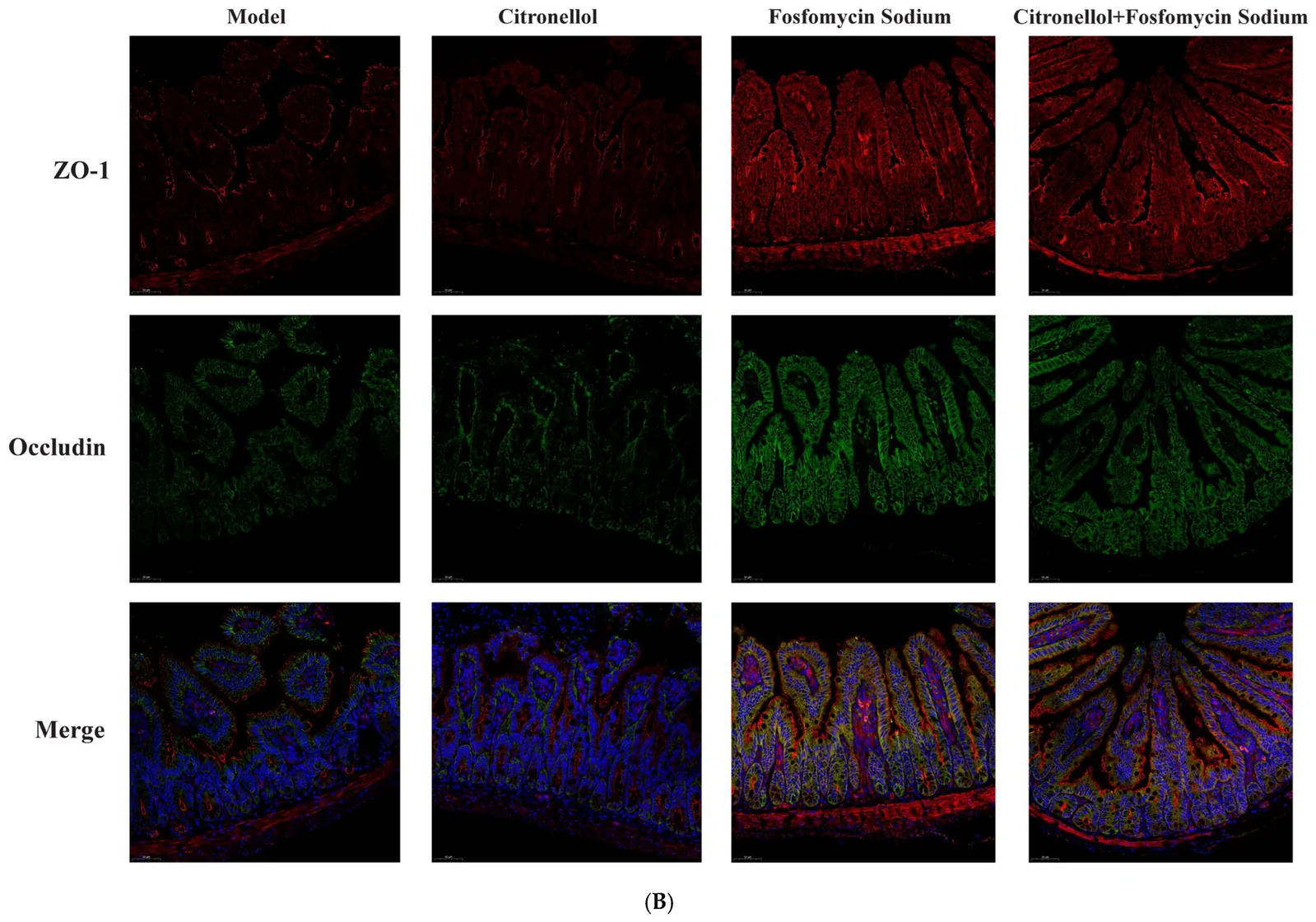
Histopathological and immunofluorescence analysis of citronellol combined with fosfomycin sodium against *E. coli* O157 infection in a murine model. (**A**) Histopathological analysis of liver, spleen, and ileum from *E. coli* O157-infected mice after treatment. Liver: red arrow, hepatic venous congestion; black arrow, inflammatory cell infiltration; orange arrow, spongy degeneration of hepatocytes. Spleen: gray arrow, dense extracellular masses formed by pigment-laden macrophages; black arrow, increasing number of immune cells; green arrow, hyperemia, congestion, or hemorrhage. Ileum: red circle, fractured intestinal villi; black circle, thinning of the muscularis layer with increased permeability; blue arrow, goblet cells in the epithelial villi. (**B**) Immunofluorescence staining of ileal tight junction proteins in *E. coli* O157-infected mice. Mice were treated with vehicle (*E. coli* O157), citronellol alone, fosfomycin sodium alone, or their combination.

Building on the enhanced antimicrobial activity observed in vitro, the protective potential of the citronellol–fosfomycin combination was evaluated in a murine model. Survival curve analysis showed a significant protective effect (Figure 5A). While fosfomycin sodium alone gave partial protection, the combination therapy markedly improved survival and rescued 80% of mice by day 7 compared to 60% with fosfomycin alone. Bacterial load analysis showed a drastic reduction in systemic organs after combination treatment. Although splenic bacterial loads were slightly lower in the combination group (Figure 5B,C), in stark contrast, hepatic bacterial burden was markedly reduced compared to all other groups (*p* < 0.01). H&E staining (Figure 6A) further validated these protective effects. In the ileum, the model group showed severe mucosal disruption, including villous atrophy, epithelial necrosis, and dense lamina propria inflammation. The combination group showed marked improvement in intestinal architecture, with preserved muscularis layer integrity, reduced epithelial permeability, and restored goblet cell morphology. The liver and spleen showed similar improvements, where the combination therapy substantially reduced diffuse inflammation and architectural damage, outperforming monotherapy. To assess the structural basis for this protection, immunofluorescence staining of tight junction proteins (Figure 6B) was performed. The model group showed reduced ZO-1 and occludin signals, indicating a damaged epithelial barrier. The combination treatment robustly restored the expression and localization of these proteins and helped rebuild the gut mucosal barrier.

## 4. Discussion

### 4.1. Citronellol Suppresses Biofilm Formation and Swimming Motility

The canonical *luxS* inhibitor furanone C30 strongly suppressed fluorescence, establishing a reliable screening method for identifying novel QS inhibitors. Using this reporter system, citronellol was identified as a potential compound that perturbs bacterial physiology in a QS-relevant context. Citronellol markedly attenuated fluorescence output in the *luxS*-eGFP reporter assay, indicating that it perturbs the physiological state of the reporter strain. The *luxS*-eGFP reporter provided an initial screen for QS modulation, and subsequent qRT-PCR (Section 3.2) confirmed citronellol’s effect on luxS transcription. Additionally, citronellol reduced bioluminescence in the *V. harveyi* BB170 reporter assay. However, biosensor toxicity or quenching by citronellol cannot be excluded as the cause of reduced luminescence. In *E. coli* O157, AI-2-mediated quorum sensing coordinates the collective bacterial behaviours required to establish and maintain complex biofilm architectures [37]. Moreover, LuxS connects AI-2 signalling to the larger regulatory network controlling virulence and motility [38]. Notably, the approximately 80% reduction in biofilm biomass at 200 μg/mL represents a strong phenotypic response, confirmed by metabolic activity assays showing no substantial effect on bacterial metabolism. The dose–response profile plateaus beyond 200 µg/mL, consistent with maximal modulation of the LuxS/AI-2 pathway at this concentration. This plateau likely reflects the upper limit of the phenotypic response. Importantly, citronellol still inhibited biofilm formation by more than 20% even at the lowest concentration (6.25 µg/mL). SEM images visually confirmed this inhibition. The control group showed dense, compact, multilayer biofilms, while citronellol-treated groups showed sparse, dismantled biofilms, indicating disrupted matrix and bacterial adhesion.

Future studies should use confocal microscopy with live/dead staining to better assess biofilm viability. Meanwhile, the impairment of swimming motility in a dose-dependent manner indicates that citronellol disrupts the core dissemination mechanism of the pathogen. Swimming requires flagella and depends on master regulators *flhC* and *flhD*, which are downstream of LuxS/AI-2 signalling [39,40]. The reduced swimming diameter likely results from modulation of this signalling axis, potentially by downregulating flagellar genes. Disrupting both biofilm and motility is a key antivirulence strategy, as it stops the pathogen from using its full set of colonisation and persistence tools. Collectively, citronellol disrupts the structural and locomotive foundations of *E. coli* O157 pathogenicity, which matches modulation of the core phenotypic outputs of the LuxS/AI-2 signalling axis. By inhibiting biofilm formation and impairing motility without affecting bacterial metabolic activity, citronellol may help the host clear the bacteria. While these findings demonstrate phenotypic disruption, they do not rule out upstream regulatory components or direct interaction with the LuxS enzyme. Future studies should use molecular docking and enzyme kinetics to find the exact molecular target.

### 4.2. Citronellol Modulates AI-2 QS and Motility Genes in E. coli O157

Transcriptional profiling provides insights into the transcriptional landscape associated with citronellol-mediated suppression of biofilm formation and motility. The data support a model in which citronellol interferes with LuxS/AI-2 signalling, potentially by affecting both AI-2 production and detection. The significant downregulation of *luxS* suggests that citronellol may reduce the intracellular production of AI-2, which could alter the capacity of bacteria for signal transduction [28]. However, this transcriptional landscape revealed a pronounced rewiring of the AI-2 QS circuitry in response to citronellol. While *luxS* expression was suppressed, indicating reduced AI-2 production, the regulatory circuitry of the *lsr* operon exhibited a marked divergence. Specifically, the regulatory and processing genes (*lsrR*, *lsrF*, and *lsrG*) were markedly upregulated, whereas the structural components of the importer (*lsrA*, *lsrB*, *lsrC*, and *lsrD*) and the initiating kinase (*lsrK*) were significantly downregulated. This expression pattern may reflect how bacteria respond to reduced intracellular AI-2. The upregulation of *lsrR*, which encodes a transcriptional repressor [41], likely acts as a feedback mechanism to prevent costly expression of the transport apparatus when intracellular AI-2 is low. This interpretation remains to be confirmed through promoter activity assays. Conversely, the induction of *lsrF* and *lsrG* could imply a continued need for processing any residual AI-2 that enters the bacterial cytoplasm [42]. However, this interpretation is speculative and requires validation via direct AI-2 quantification. This coordinated downregulation of the lsrACDB transporter complex [43] and *lsrK* [44] suggests impaired uptake and phosphorylation, consistent with attenuated QS signal transduction. Downregulating *lsrK* may impair AI-2 phosphorylation, which would keep the *lsr* operon repressed. Collectively, these transcriptional changes align with reduced AI-2 signalling in the bacterial population. However, these interpretations are limited by the lack of direct AI-2 quantification and promoter-level validation, and further functional studies are needed.

The repression of *csgD*, the master regulator of biofilm matrix production [45], explains the dismantling of biofilm architecture observed via SEM. As the transcriptional activator *csgD* controls the expression of curli (*csgA*) and cellulose (*bcsA*), its downregulation may limit these adhesive and structural components essential for biofilm formation [46]. Similarly, the strong suppression of the flagellar master regulators *flhC* [47] and *flhD* [48] is consistent with the impaired swimming motility. The flagellar cascade, which includes basal body assembly and the motor complex genes *motA* and *motB* [49], was markedly reduced. The lower expression further supports the loss of motile capability. Collectively, these findings suggest that citronellol is a QS modulator that alters key virulence-associated phenotypes. However, although the data suggest interference at multiple QS nodes (*luxS*, *lsr* operon, *csgD*, and *flhDC*), the exact target and upstream cascade need further study. The suppression of both biofilm formation and motility suggests that citronellol may interact with broader regulatory networks, such as those controlled by second messengers like c-di-GMP [50].

### 4.3. Combination Effects of Citronellol with Antibiotics

The combinatorial screening showed that citronellol augments the activity of conventional antibiotics against *E. coli* O157 across multiple classes, enhancing their antibacterial effects at sub-inhibitory concentrations. This enhanced activity was most pronounced with fosfomycin, azithromycin, gentamicin, and streptomycin (Figure 4A–D), where citronellol reduced bacterial viability at concentrations as low as 1/8 MIC. The enhancement was concentration-dependent. For antibiotics such as chloramphenicol, tetracycline, and cefotaxime acid (Figure 4E–G), citronellol did not enhance activity at 1/2 MIC, but the effect appeared at 1/4 and 1/8 MIC. This phenomenon may reflect the induction of tolerant persister states at higher sub-MICs [51]. At 1/2 MIC, these antibiotics exert enough selective pressure to trigger strong bacterial stress responses. These responses likely cause damage control and metabolic rewiring, often at the cost of communal behaviours, potentially hiding the anti-biofilm effect of citronellol or redirecting resources away from QS regulation. Conversely, at 1/4 and 1/8 MIC, the pressure may be insufficient to initiate these global protective programmes. This allows citronellol to disrupt QS signalling and biofilm architecture, sensitizing the otherwise tolerant population. This suggests that lower dose combinations may be the best strategy for maximising efficacy while minimising resistance risk. Polymyxin B was the only exception. Unlike fosfomycin, gentamicin, and azithromycin, citronellol did not enhance the activity of polymyxin B. This may partly reflect the distinct cellular target of polymyxin B [52], but other factors likely contribute, including differences in antibiotic class, existing resistance mechanisms, and drug penetration into biofilms. A comprehensive evaluation of these variables will be required to fully define the determinants of adjuvant–antibiotic compatibility. In summary, citronellol acts as a potent antimicrobial adjuvant with its combination activity spanning diverse antibiotic classes except polymyxin B. This enhanced activity is closely associated with QS modulation by citronellol, which disrupts biofilm architecture and may attenuate the risk of resistance evolution. Collectively, these data support the continued development of citronellol-based combination strategies for *E. coli* O157 and indicate its protective potential in mitigating biofilm-associated infections in vivo.

### 4.4. Efficacy of Citronellol in Combination with Fosfomycin Sodium

The enhanced in vitro activity translated to the murine systemic infection model, supporting citronellol as a promising antivirulence adjuvant. The citronellol and fosfomycin combination gave 80% survival, a significant improvement over the 60% with fosfomycin alone, matching a model where LuxS/AI-2 signalling modulation and altered biofilm dynamics improve outcomes in vivo. Lower systemic bacterial burden, especially in the liver, drove this survival benefit. Consistent with its in vitro effects, citronellol may disrupt the physical barrier restricting antibiotic penetration, potentially improving fosfomycin accessibility to intracellular and matrix-embedded bacteria within the hepatic parenchyma. Histopathological analysis further supported this protective effect. The combination group showed restored intestinal architecture with intact, elongated villi and no inflammatory infiltrate, unlike the severe villous disruption in the model group. This structural preservation matched the restoration of tight junction proteins (ZO-1 and Occludin), suggesting that citronellol may mitigate intestinal hyperpermeability [53,54]. Although these findings show the preclinical potential of this combination, they are limited to female C57BL/6J mice and a 7-day observation. Future studies should test this adjuvant strategy with other antibiotic classes, use systematic dose–response analyses, and include both sexes to define its broader utility.

In summary, citronellol modulates the LuxS/AI-2 QS axis and enhances the efficacy of fosfomycin against *E. coli* O157 in vivo. However, whether citronellol directly interacts with the LuxS active site requires biochemical validation. Furthermore, reliance on a single reference strain and the absence of comprehensive toxicological assessments limit the broader applicability of our conclusions. Future investigations should prioritize elucidating the molecular target using biophysical assays, validating efficacy across diverse clinical isolates and immunocompromised models, and establishing a robust safety margin through systematic toxicology.

## 5. Conclusions

This study identifies citronellol as a promising agent against *E. coli* O157, with its effects closely associated with modulation of the LuxS/AI-2 signalling axis. Citronellol reduced reporter activity in the *V. harveyi* BB170 bioassay, which correlated with downregulation of *luxS* expression and alterations in the *lsr* operon transcriptional profile. Importantly, these effects occurred without harming bacterial metabolic activity. This supports the potential of citronellol to act as an adjuvant that enhances conventional antibiotics at doses below the MIC. In a mouse systemic infection model, the citronellol and fosfomycin combination improved survival and reduced liver bacterial burden, confirming its protective potential.

Although these in vivo benefits are consistent with LuxS/AI-2 modulation observed in vitro, a definitive causal link within the host environment remains to be established. Collectively, this work makes three key advances. First, it is the first to show that citronellol impairs *E. coli* O157 biofilm formation, likely by disrupting the quorum sensing system. Second, citronellol acts as an adjuvant that not only reduces selective pressure for resistance development but also augments the antibacterial activity of certain antibiotics. Third, this study provides direct in vivo evidence that citronellol combined with specific antibiotics like fosfomycin enhances protection against *E. coli* O157 infection, establishing an adjuvant strategy for multidrug-resistant infections. Nevertheless, further work is needed to fully characterize the molecular basis of these observations. Future studies, including molecular docking, enzyme kinetic analyses, and validation across diverse clinical isolates, will be essential to define its mechanism of action and support the development of new antimicrobial agents.

## Data Availability

The data described in this study are accessible upon request from the corresponding author.

## Supplementary Materials

The following supporting information can be downloaded at: https://www.mdpi.com/article/10.3390/biom16101439/s1, Table S1: Primer sequences for PCR analysis; Table S2: The Primer sequences for qRT-PCR; Figure S1: The chemical structure of citronellol; Figure S2: Growth curves (CFU) of *E. coli* O157 treated with different concentrations of citronellol (50 µg/mL, 100 µg/mL, and 200 µg/mL) or untreated (Control). No significant difference was observed between the treatment and control groups. Data are presented as mean ± SD (*n* = 3); Figure S3: Schematic map of the pUC19-*luxS*-eGFP recombinant plasmid.
