# Supplementary material for: Citronellol Potentiates Antibiotics Against Escherichia coli O157:H7 via Inhibition of Biofilm Formation
Source: Biomolecules. 2026 Oct 2;16(10):1439. doi: 10.3390/biom16101439 (PMC13702520; doi:10.3390/biom16101439)
Supplement: Supplementary file 1 [file biomolecules-16-01439-s001.zip › biomolecules-4560635-supplementary.pdf]

Supplementary Material

Table S1. Primer sequences for PCR analysis

Target genes		Sequences (5'-3')
luxS	Forward	GGATCCTCTAGAATGCCGTTGTTAGATAGCTTCACAG
	Reverse	TGCTCACCCTCCGATGTGCAGTTCCTGCAACTTCTC
luxS-eGFP	Forward	TACGCCAAGCTTATGCCGTTGTTAGATAGCTTCACAG
	Reverse	GGATCCTCTAGATTACTTGTACAGCTCGTCCATGCC

Table S2. The Primer sequences for qRT-PCR

Target genes		Sequences (5'-3')
luxS	Forward	GAAAACAATGAACACCCCGCATGG
	Reverse	TCCCTCTTTCTGGCATCACTTCTTTG
csgD	Forward	AATCGCTGGCAATTACAGG
	Reverse	CCGCTTCCATCATATCCAG
flhC	Forward	ATGCTGCCATTCTCAACCGACTG
	Reverse	CGCATCGACGCCATTACACAAAC
flhD	Forward	CGTTAGCGGCACTGACTCTTCC
	Reverse	TTGCGTCAACTGAGTAATCGTCTGG
lsrA	Forward	ATGCAAACGAGTGATACCCGCGC
	Reverse	ACTTCAGCATGACGCCTCCTGAC
lsrB	Forward	AGTGCTGACCTGGGACTCTGATAC
	Reverse	GCCATATCCACCAACATACCTCCTAAC
lsrF	Forward	TCGGCAGCGAATATGAACATCAGTC
	Reverse	CCATATCTTTGCCCACGCCAGTC
lsrK	Forward	GATGAACCTACCGCCTCGCTTAC
	Reverse	AACAATACCCACGCCAGTAGCAAG
lsrC	Forward	GACGATAATTCTGGTGGCATTTATGGC
	Reverse	TGCGAATGGCTTCAGTACGAACG
lsrD	Forward	GTTTCAAAGTGGTGTGCCGATGC
	Reverse	AGATACAGCGTGCCAAGCGTAATC
lsrG	Forward	AAGCCTATAAAGATGAAGACGCAGTGG
	Reverse	CACGGCATCAAACCATTGAACAGAC
lsrR	Forward	ACCACAACAGATGCTGGCGATTG
	Reverse	GCTGCCCGATTCCCGTCATATAAG
motA	Forward	GAAGCCTTGGAGCACTCTATCAACC
	Reverse	CTTTGGTGTATTTGGAGCGACGAAAC
motB	Forward	CAGTGCCGATGTCGAACCCTATATG
	Reverse	GCTCCCAGTTGCTATATCCTTTCTCAC

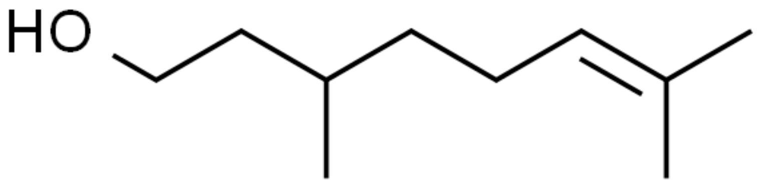

Figure S1. The chemical structure of citronellol.

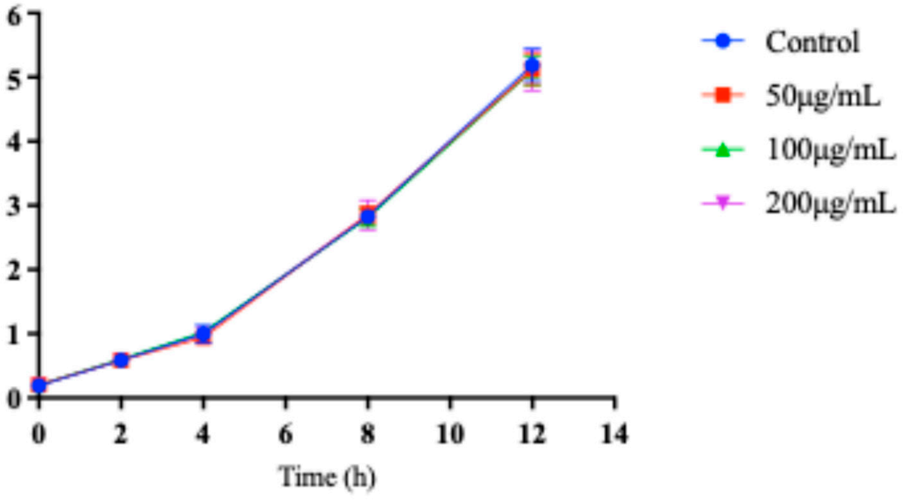

Figure S2. Growth curves (CFU) of *E. coli* O157 treated with different concentrations of citronellol (50μg/mL, 100μg/mL, and 200μg/mL) or untreated (Control). No significant difference was observed between the treatment and control groups. Data are presented as mean ± SD (n=3).

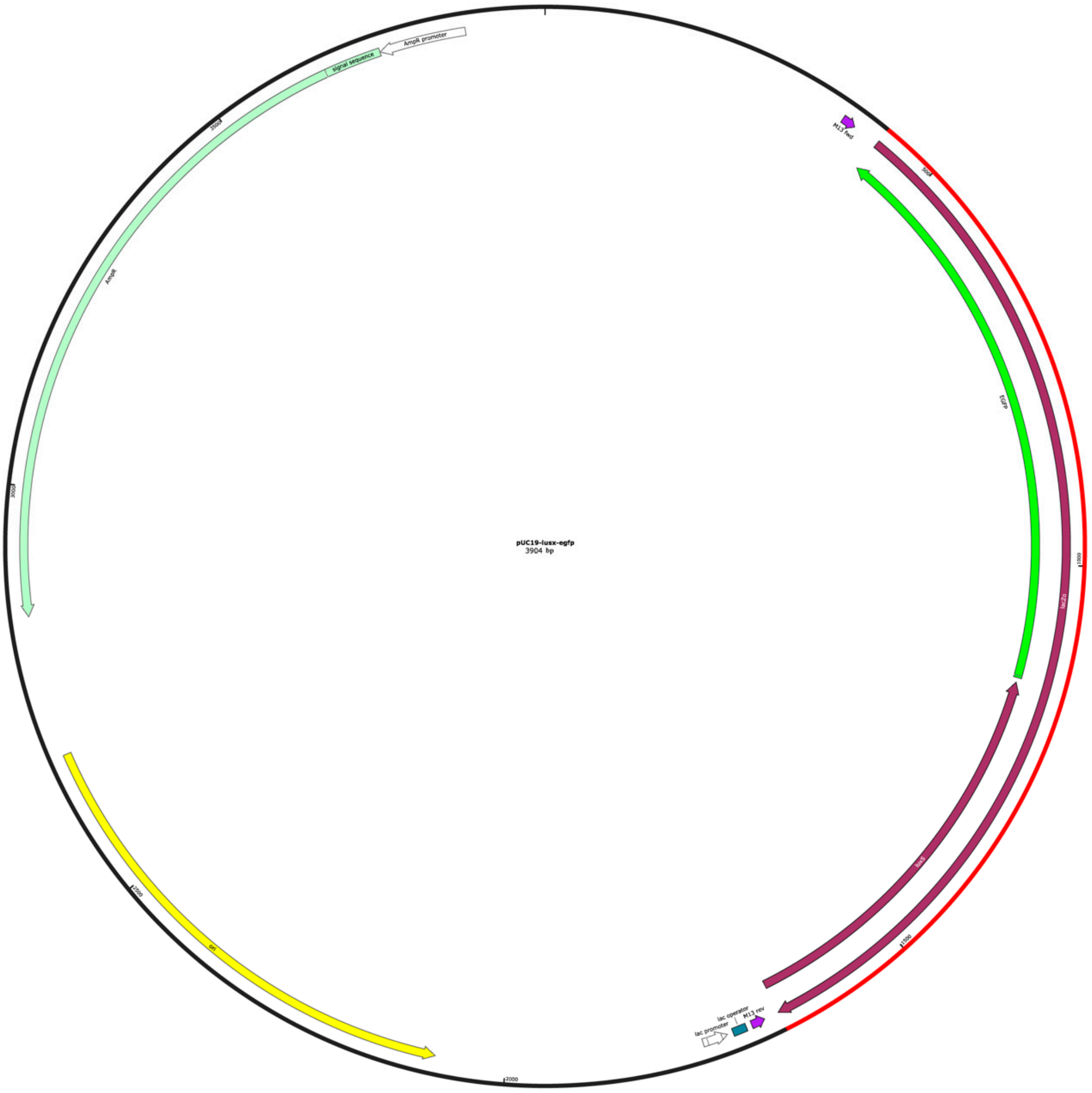

Figure S3. Schematic map of the pUC19-*luxS*-eGFP recombinant plasmid.
